# Preserving Reproductive Potential in the Solitary Testis With Germ Cell Tumour and Azoospermia Using Onco-Testicular Sperm Extraction (TESE): A Case Report

**DOI:** 10.7759/cureus.80919

**Published:** 2025-03-20

**Authors:** Obaidullah Durrani, Umar Arif, Hassan Baig, Tarek Khalil, Ghulam Nandwani

**Affiliations:** 1 Department of General Surgery, Ninewells Hospital, Dundee, GBR; 2 Department of General Surgery, Queen Elizabeth University Hospital, Glasgow, GBR; 3 Department of Urology, Ninewells Hospital, Dundee, GBR

**Keywords:** andrology, azoospermia, fertility preservation, germ cell tumours (gct), male fertility, onco-tese, testicular sperm extraction (tese)

## Abstract

Patients with germ cell tumours in solitary testis have significantly reduced fertility potential. Preserving fertility is crucial for improving quality of life and providing patients with the opportunity to conceive in the future if desired. This study introduces a novel technique for fertility preservation in patients who would otherwise face infertility.

We describe a novel surgical technique in a 29-year-old patient with a germ cell tumour in the solitary testis and a history of previous orchidectomy for undescended testis. On-table extraction of viable spermatozoa was performed during tumour excision to preserve the patient’s reproductive potential. Spermatozoa and testicular tissue samples were sent for histopathological analysis to determine the feasibility of extraction. The pathology report confirmed the presence of normal testicular tissue within the affected testis, allowing for the successful extraction of viable spermatozoa. These spermatozoa were subsequently used for successful oocyte fertilisation via intracytoplasmic sperm injection (ICSI).

This technique enables the extraction of viable spermatozoa from patients with a germ cell tumour in the solitary testis. It offers a viable fertility preservation strategy for male patients undergoing orchidectomy or partial orchidectomy with adjuvant chemoradiotherapy. Additionally, this approach enables histopathological confirmation of spermatozoa viability before radical orchidectomy, optimising fertility preservation.

## Introduction

Testicular germ cell tumours (GCTs) are the most common solid neoplasms in males aged 15-35 years [1]. Fertility preservation is a crucial consideration in young patients, many of whom have not yet started or completed their families. Studies show that more than 50% of patients with GCTs have oligospermia, while 6%-24% present with azoospermia. Furthermore, adjuvant chemotherapy, surgery, and radiotherapy contribute to azoospermia in up to 60% of patients [2,3].

Currently, semen cryopreservation before anti-cancer therapy remains the standard method for fertility preservation. However, this approach is only viable if viable spermatozoa are present in the ejaculate, making it unsuitable for azoospermic patients. In non-GCT cases, testicular sperm extraction (TESE) or percutaneous sperm aspiration can be used to retrieve spermatozoa before definitive oncological treatment, provided there is adequate time and access to specialised facilities [4,5].

Fertility preservation becomes particularly challenging in patients with a tumour in the solitary testis, bilateral testicular tumours, or severe contralateral testicular atrophy. In such cases, the urgency of oncological treatment often takes precedence over fertility preservation. To address this, onco-TESE, defined as TESE performed during radical orchidectomy, offers a potential solution. Here, we present a case of onco-TESE performed in an azoospermic patient with a solitary testis affected by GCT, demonstrating its feasibility as a fertility preservation strategy in such complex cases.

## Case presentation

A 29-year-old male patient with a history of unilateral orchidectomy 10 years prior for an undescended testis presented for investigation of azoospermia. Examination revealed normal secondary sexual characteristics and no abnormalities in the remaining solitary testis. However, a biopsy of the testis demonstrated atrophic seminiferous tubules, many containing only Sertoli cells, with no evidence of active spermatogenesis. Intratubular germ cell neoplasia was also identified. The patient was subsequently placed under active surveillance.

**Figure 1:**
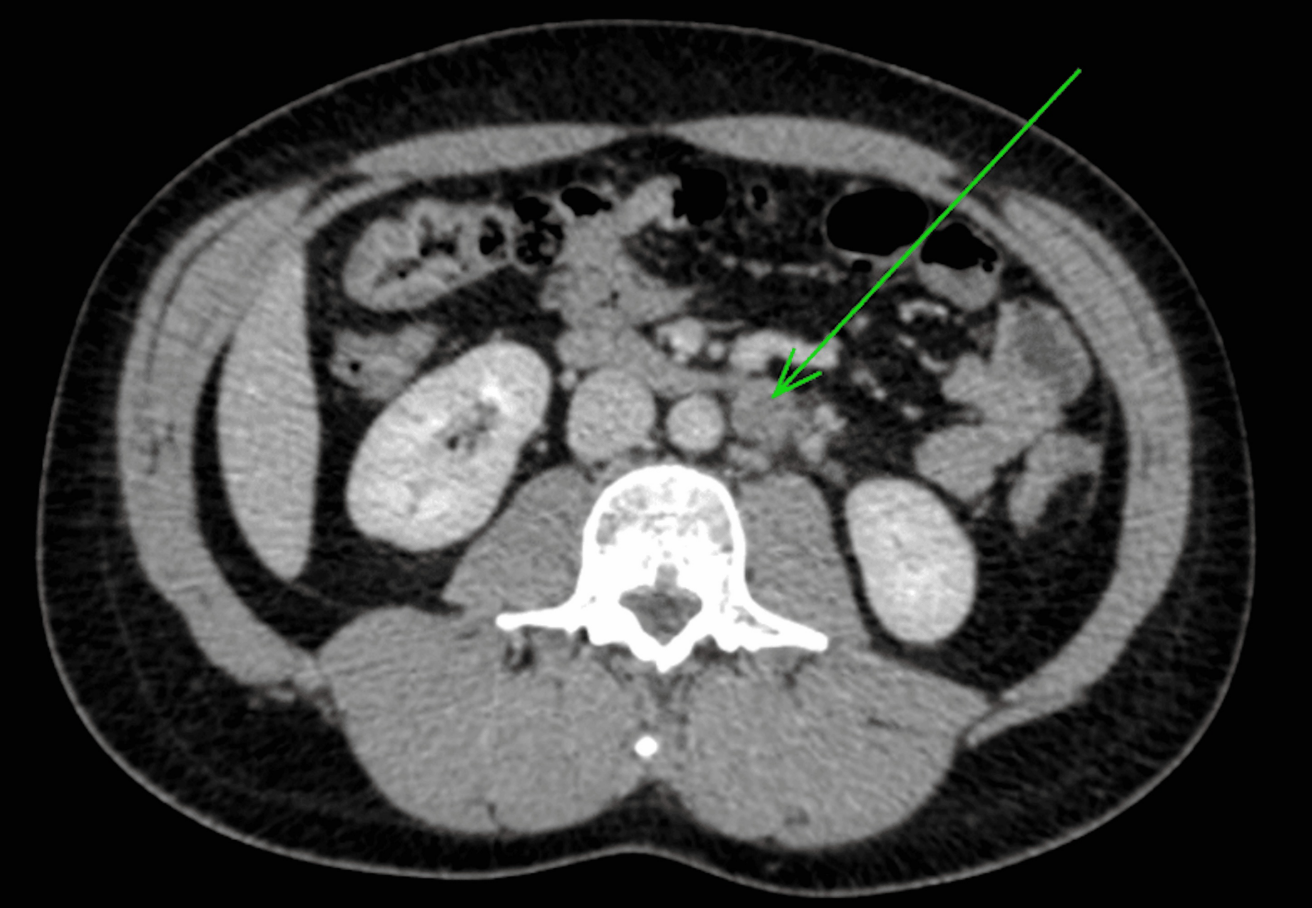
Axial computed tomography scan of the abdomen showing para-aortic lymphadenopathy

**Figure 2:**
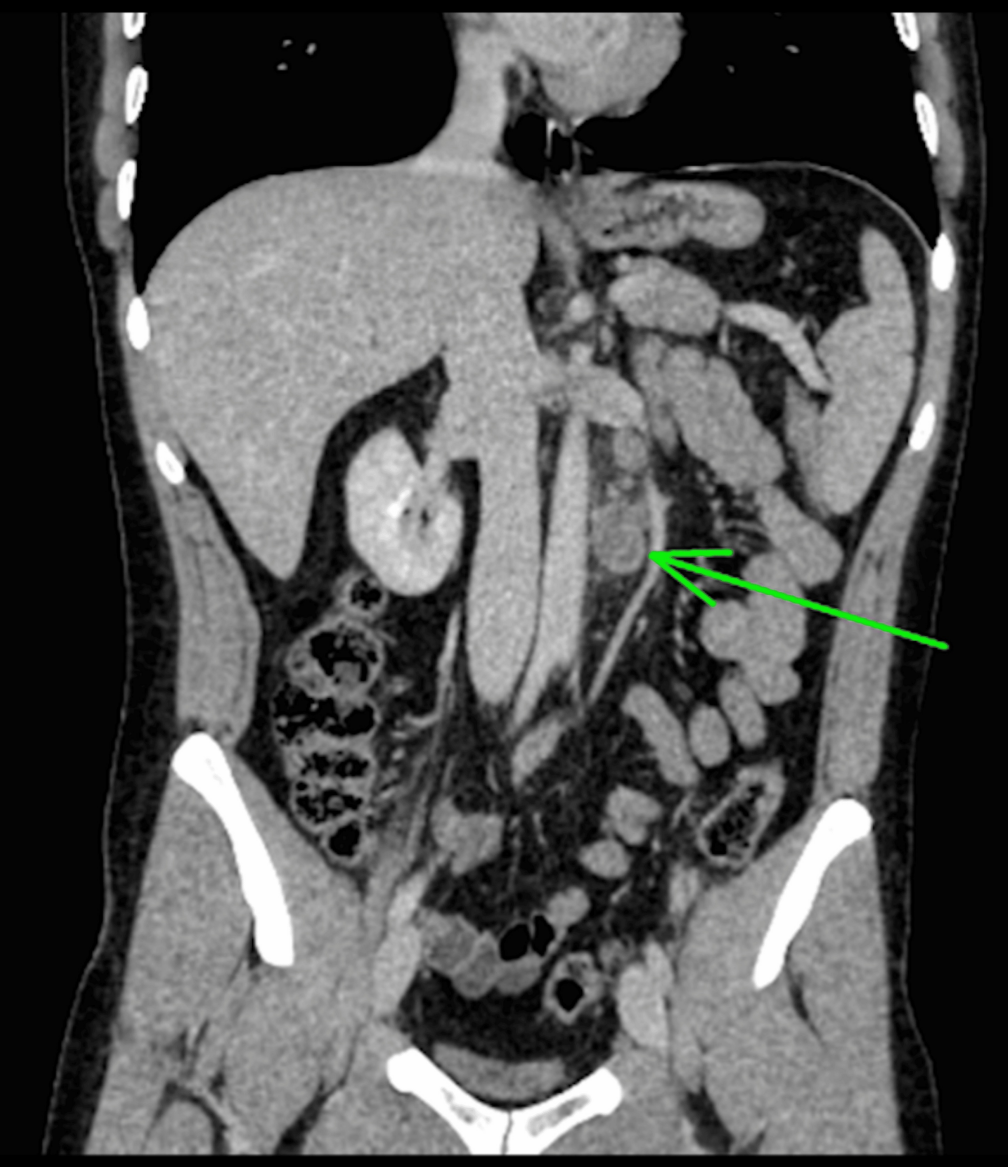
Coronal computed tomography scan of the abdomen showing para-aortic lymphadenopathy

Six months later, he experienced discomfort in the remaining testis and noticed a progressively enlarging mass on self-examination. Subsequent ultrasound assessment identified a hypoechoic lesion with characteristics strongly indicative of a GCT. A staging CT scan of the chest, abdomen, and pelvis detected a 2 cm para-aortic lymphadenopathy (Figures 1, 2).

**Figure 3:**
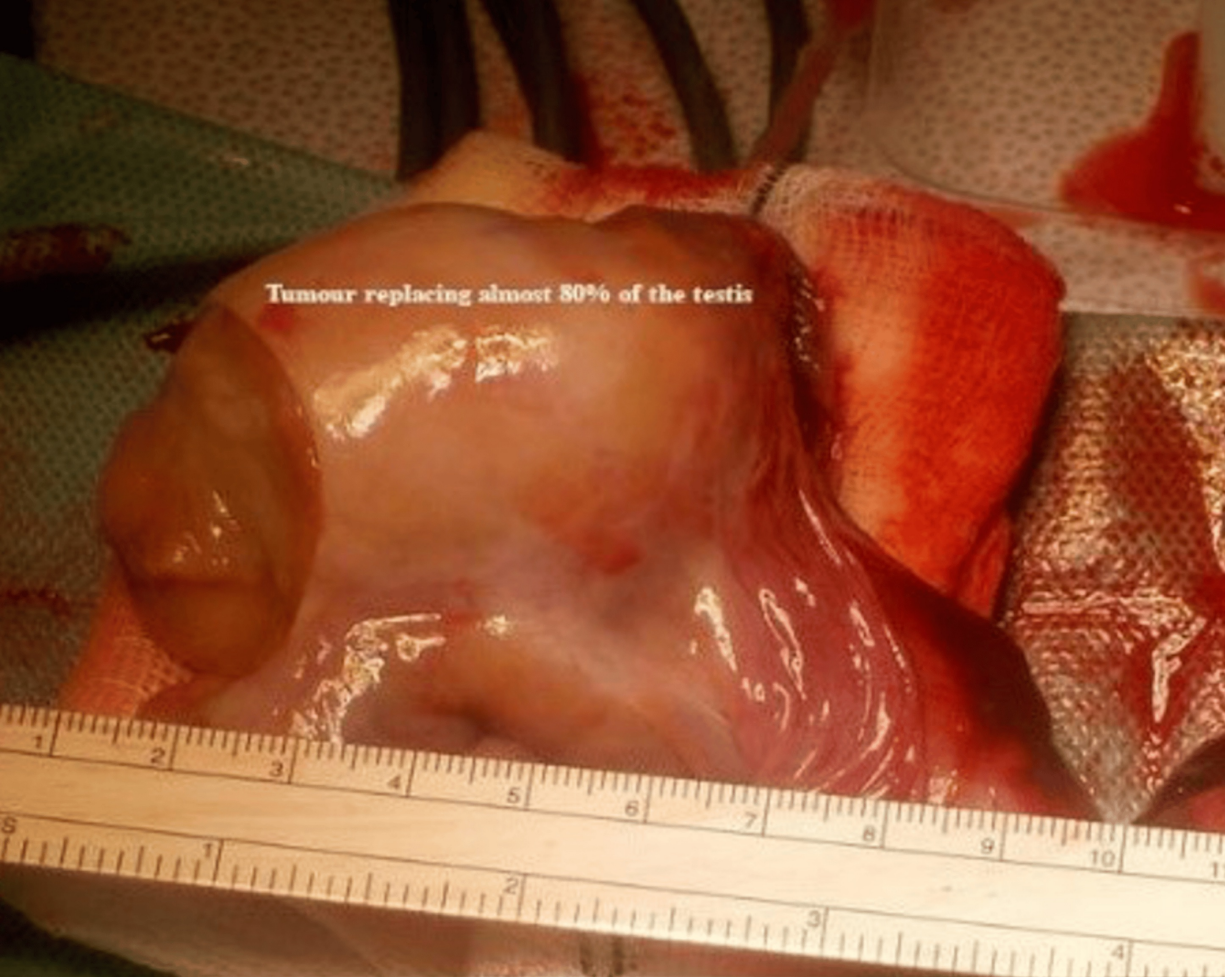
Testicular germ cell tumour occupying 80% of the testis

Serum tumour markers, including alpha-fetoprotein and human chorionic gonadotropin, were within normal limits, but lactate dehydrogenase was significantly elevated at 272 µmol/L. After discussing the findings with the patient, a radical inguinal orchidectomy was performed. Intraoperatively, the tumour was found to occupy more than 80% of the testicular parenchyma (Figure 3).

**Figure 4:**
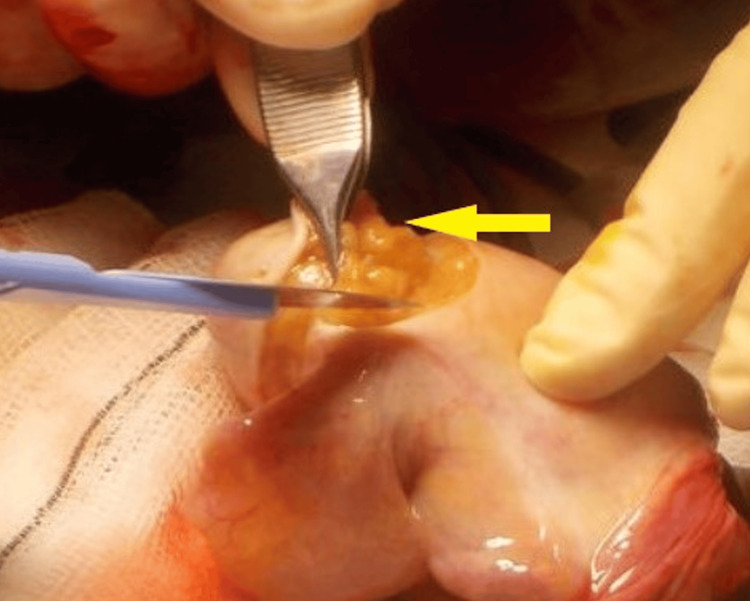
Intraoperative image showing seminiferous tubules isolated during bench dissection for onco-TESE TESE: testicular sperm extraction

**Figure 5:**
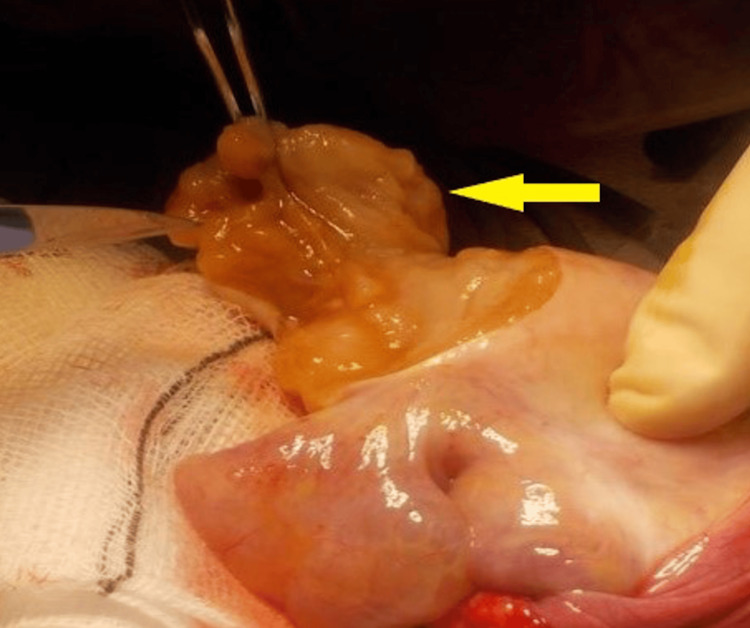
Intraoperative photograph demonstrating seminiferous tubules identified during bench dissection of the excised testis for sperm retrieval

Bench dissection of the macroscopically normal-appearing one-third of the testis was carried out to identify seminiferous tubules (Figures 4, 5).

A specimen was sent to the laboratory, where spermatozoa with some motility (grade C) were retrieved. The sperm concentration was <1 million per millilitre, and the collected spermatozoa were cryopreserved in eight vials. Following successful sperm retrieval, the entire testis was sent for histopathological examination. The tumour was confirmed as a 5.5 cm mixed GCT (40%-50% classical seminoma and 50%-60% embryonal carcinoma), confined to the testis with extensive lymphovascular invasion into the cord. The final Royal Marsden stage was 2A (tumour, node, metastasis (TNM): T2 N2 M0 S0). The patient subsequently received three cycles of bleomycin, etoposide, and cisplatin with curative intent. At his most recent follow-up, 36 months post-procedure, he remained free of recurrence.

The patient and his partner proceeded with intracytoplasmic sperm injection (ICSI), resulting in successful oocyte fertilisation. However, implantation failure occurred due to an unreceptive endometrium, leading to the decision not to proceed with a clinical pregnancy at that time. Further in vitro fertilisation cycles were planned. Importantly, the extraction of viable spermatozoa enabled successful fertilisation, demonstrating the feasibility of onco-TESE in fertility preservation.

## Discussion

In patients of reproductive age diagnosed with GCTs, fertility preservation is a critical aspect of maintaining quality of life. Current guidelines recommend that fertility preservation options be discussed at the earliest opportunity [6]. Sperm banking through semen collection is a widely used and straightforward cryopreservation technique. However, this method may not be suitable for patients with prior ejaculatory disorders, severe oligozoospermia, or azoospermia. In such cases, TESE provides a reliable alternative and can be performed before the initiation of chemotherapy or radiotherapy [7]. Onco-TESE involves retrieving spermatozoa from normal testicular tissue prior to commencing chemotherapy or radiotherapy. This approach has been successfully used in patients with azoospermia, severe oligospermia, ejaculatory disorders, and solitary testes, without delaying definitive cancer treatment [8].

Existing literature suggests that onco-TESE has been performed in a limited number of patients, with a reported success rate of slightly over 50%. However, its use in patients with a solitary testis undergoing orchidectomy for either malignant or benign pathology has only been described in six cases [9-11]. Successful sperm retrieval was achieved in five of these cases, confirming the feasibility of this technique.

Testicular tumour size is inversely correlated with spermatogenesis and Johnson’s score. Spermatogenesis is more preserved in seminiferous tubules located away from the tumour. Patients with large tumours often have reduced viable testicular tissue, making sperm retrieval more difficult [12]. However, successful sperm extraction has been documented in tumours up to 7 cm in size, even when nearly the entire testis is affected [13]. In our case, the tumour measured 5.5 cm and occupied two-thirds of the testis, yet viable spermatozoa were successfully retrieved.

Our patient had a prior orchidectomy and was already suffering from infertility due to azoospermia. Pre-diagnosis TESE was unsuccessful, and radical orchidectomy of the solitary testis was unavoidable. By performing onco-TESE intraoperatively and identifying normal testicular tissue during bench dissection, fertility potential was preserved in this patient, despite the need for definitive oncological treatment.

## Conclusions

This case demonstrates the feasibility of onco-TESE as a fertility preservation strategy in patients with a solitary testis affected by a large GCT. Despite the patient’s pre-existing azoospermia and extensive tumour involvement, meticulous bench dissection enabled the retrieval of viable spermatozoa, which were successfully used for ICSI.

The findings underscore the inverse correlation between tumour burden and spermatogenesis, highlighting the importance of precise identification of residual functional seminiferous tubules for sperm retrieval. Onco-TESE allows for concurrent histopathological assessment and sperm extraction without delaying definitive oncological treatment. Given its potential to preserve reproductive potential in otherwise infertile patients, onco-TESE should be considered as a standard adjunctive procedure during radical orchidectomy in select cases. Further research is warranted to refine the technique, optimise sperm retrieval rates, and improve clinical outcomes for assisted reproduction in this patient population.
